# Tracker-Based Personal Advice to Support the Baby’s Healthy Development in a Novel Parenting App: Data-Driven Innovation

**DOI:** 10.2196/12666

**Published:** 2019-07-24

**Authors:** Renée A Otte, Alice J E van Beukering, Lili-Marjan Boelens-Brockhuis

**Affiliations:** 1 Philips Research Family Care Solutions Eindhoven Netherlands; 2 Philips Consumer Lifestyle Mother & Childcare Eindhoven Netherlands

**Keywords:** data analytics, data-driven science, mHealth, mobile apps, infant development, infant health, parenting

## Abstract

**Background:**

The current generation of millennial parents prefers digital communications and makes use of apps on a daily basis to find information about child-rearing topics. Given this, an increasing amount of parenting apps have become available. These apps also allow parents to track their baby’s development with increasing completeness and precision. The large amounts of data collected in this process provide ample opportunity for data-driven innovation (DDI). Subsequently, apps are increasingly personalized by offering information that is based on the data tracked in the app. In line with this, Philips Avent has developed the uGrow app, a medical-grade app dedicated to new parents for tracking their baby’s development. Through so-called insights, the uGrow app seeks to provide a data-driven solution by offering parents personal advice that is sourced from user-tracked behavioral and contextual data.

**Objective:**

The aim of this study was twofold. First, it aimed to give a description of the development process of the insights for the uGrow app. Second, it aimed to present results from a study about parents’ experiences with the insights.

**Methods:**

The development process comprised 3 phases: a formative phase, development phase, and summative phase. In the formative phase, 3 substudies were executed in series to understand and identify parents’ and health care professionals’ (HCPs) needs for insights, using qualitative and quantitative methods. After the formative phase, insights were created during the development phase. Subsequently, in the summative phase, these insights were validated against parents’ experience using a quantitative approach.

**Results:**

As part of the formative phase, parents indicated having a need for smart information based on a data analysis of the data they track in an app. HCPs supported the general concept of insights for the uGrow app, although specific types of insights were considered irrelevant or even risky. After implementing a preliminary set of insights in a prototype version of the uGrow app and testing it with parents, the majority of parents (87%) reported being satisfied with the insights. From these outcomes, a total of 89 insights were implemented in a final version of the uGrow app. In the summative phase, the majority of parents reported experiencing these insights as reassuring and useful (94%), as adding enjoyment (85%), and as motivating for continuing tracking for a longer period of time (77%).

**Conclusions:**

Parents experienced the insights in the uGrow app as useful and reassuring and as adding enjoyment to their use of the uGrow app and tracking their baby’s development. The insights development process we followed showed how the quality of insights can be guaranteed by ensuring that insights are relevant, appropriate, and evidence based. In this way, insights are an example of meaningful DDI.

## Introduction

### New Parents

The transition to parenthood, from being partners to also being parents, often represents an exciting but challenging phase. Most new parents are looking for knowledge about and advice on how to best take care of the new family member [1]. They might experience a need for more information when it comes down to the practical side of caring for an infant. Learning how to read their infants, interpret what their needs are, and correctly act upon this can feel as daunting tasks [2-4]. This was shown by, for example, Moran et al [5], who studied primipara and multipara women. They investigated on which baby care– and self-care–related topics these mothers desired information for after childbirth. Of the first-time mothers in the sample, up to 87% liked to receive more baby-care information, for example, on baby’s illness, baby’s schedule, and on how to soothe their baby when their baby was crying.

### Changing Ways of Finding Parenting Information

Traditionally, parents have turned to their own parents or other close relatives and friends for information about infant care and parenting. However, over the last few decades, parents have found this traditional source of information increasingly difficult to rely on. First of all, because of globalization and increased mobility of the world population, young parents may live across the country, or even abroad, and thus they may be physically further removed from their loved ones than 30 to 40 years ago [6,7]. Moreover, as a study by O’Connor & Madge [1] showed, young parents experienced the child-rearing knowledge of their parents to be out of date and not reflecting current childcare practices. Therefore, nowadays, new parents often turn to other sources of information, such as books and the internet [1,6,8-11].

### New Trends for Finding Parenting Information

Another trend is the widespread use of mobile phones, which enables new parents to look up child-rearing information even quicker. With their capacity to install and launch countless apps, mobile phones contribute to a lifestyle in which the population is always on the Web [12]. This especially holds for the so-called Millennial Generation that refers to the group of individuals who were born between 1981 and 1999. When growing up, a majority of them had access to personal computers, pagers, or cell phones. They are thought to enjoy utilizing technologies and have high expectations of the usefulness and availability of these technologies in all settings [13]. Millennial parents have been found to prefer digital communications (eg, through Facebook, email, and short message service text messages) [14] and make use of apps on an almost daily basis to find information about caring for their infants [15,16].

### Parenting Apps

Unsurprisingly, more and more parenting apps have become available to support new parents; a quick search for parenting apps in the Google Play Store easily yields more than 50 hits within a second. These apps are becoming ever smarter, in that they do not only contain information about child-rearing topics but they also allow users to track the infants’ development with increasing completeness and precision [16]. The often large amounts of contextual and behavioral data (referred to as big data) that are collected in (and in function of) the process provide ample opportunity for data-driven innovation (DDI) [17]. By means of different techniques—artificial intelligence, machine learning, data mining, and classification algorithms—the collected contextual and behavioral data can be analyzed and turned into meaningful, new information for the infant’s parents or caregivers [18,19]. Given this, more apps are increasingly personalized by offering tailored information and personal advice on the basis of tracked app data [20]. Moreover, according to Johnson [20], apps are designed to be performative, that is, they induce or elicit users to act, for example, to change certain behaviors.

### Insights

In line with these trends, Philips Avent has developed the uGrow app, a medical grade app dedicated to new parents for tracking their baby’s development. It seeks to provide a data-driven solution by offering parents personal advice that addresses their needs and wishes, on the basis of behavioral and contextual data users have tracked in the app. To achieve this, the uGrow team has developed so-called insights. Insights refer to small pieces of text that are shown in the uGrow app on the basis of a technical rule. This rule analyses the data that parents have tracked in the app and defines when an insight text is presented to the user. For example, if a mother is keeping track of her baby’s breastfeeds, an insight could be the following: *based on your tracked data, your left breast appears to steal the spotlight a little*. In this way, insights provide personal advice, tips, and information tailored to the unique situation of the parent and the baby. In Multimedia Appendix 1 a screenshot of uGrow can be found including an example of an insight.

### Evidence-Based

In addition to being personal, the insights are evidence-based, that is, content is based on scientific publications, guidelines of prominent institutions, such as the American Academy of Pediatrics (AAP), and discussions with experts. This is an important requirement, as research has shown that only few apps contain information that corresponds to guidelines or are supported by scientific evidence. For example, a systematic review of quality of information on infant feeding apps showed that the information studied lacked credibility and reliability [21-26]. This lack of a solid evidence base can lead to misinforming users in the best case, and it can lead to adverse health outcomes in the worst [25,26]. Thus, to ensure quality and prevent providing parents with incorrect or unsubstantiated information, we set up and adhered to a controlled and systematic development process for the insights.

### Goals of This Study

The aim of this study was twofold. The first was to give a description of the development process of insights for the uGrow app, that is, we reported on the steps that were taken from defining parents’ and health care professionals’ (HCPs) needs to the development of high-quality insights supporting parents in their parenting role. The second was to present results from a user study about parents’ experiences with the insights and their opinion on whether insights are supportive, insightful, and help reassure that their infants are developing in a healthy way.

## Methods

### Study Phases

This study comprised 3 phases: a formative phase, development phase, and summative phase. The study started with a formative phase that had 2 goals: (1) understanding the needs and context of the primary target group—parents—and (2) understanding what HCPs think of an app for parents with which parents can track their baby’s development. After the formative phase, the insights were created during the development phase. Subsequently, in the summative phase, these insights were validated against parents’ experience. As mentioned in the introduction of this paper, this study took place in the context of the overall development process of the Philips Avent uGrow app.

### Formative Phase

In the formative phase, 3 studies were executed in series to understand and identify parents’ and HCPs’ needs for insights: a Web-based community study, a study with HCPs and professionals, and an in-home user test. Each of these studies is described below.

#### Web-Based Community

The Web-based community study was executed to understand the role apps have for parents (to be) who want to track their baby’s development. Parents (or parents-to-be) eligible for participation in the Web-based community either had a baby between 0 and 12 months old or were in the third trimester of their pregnancy, were interested in the Philips My Baby and Me app—the predecessor of the uGrow app—and were frequent app users. The parents (to be) received compensation for the time they spent on study participation. The Web-based community comprised a private website environment that was custom made for the purpose of this study. Participants of the study were invited to this website environment, in which they could access both shared and private pages. On the shared pages, the community moderator could, for example, ask open-ended questions or poll questions. The participants could answer these while interacting with each other and while engaging in group discussions. For example, participants were asked what the *baby’s healthy development* means for them, and they were asked to upload photos of how they were tracking their baby’s development at the time. On the private pages, the community moderator could ask participants individual questions or give them creative tasks. For example, the moderator could ask participants to rate the importance of tracking their baby’s sleeping behavior on a 5-point Likert scale or rank questions (eg, *How much milk does my baby drink from his/her bottle every day?*) they have regarding parenting from most to least important. Thus, the Web-based community used a mixed-methods design, encompassing a qualitative and quantitative approach by combining Web-based focus group discussions, in-depth interviewing, and individual and group surveys. Through a screening process, parents were selected for participation in the Web-based community. After screening, all participants went through several steps, starting with the creation of an individual user profile, followed by warm-up discussions, polls, and individual tasks, and ending with follow-up discussions. Participants were able to communicate through text and visuals (eg, pictures, interactive visual tasks, and drawings). The data collected in the Web-based community comprised qualitative data, such as written text and pictures, and quantitative data, such as rating scale data. The qualitative data were analyzed using an inductive, qualitative analysis method [27], where the researchers coded and categorized the data to discover and define overarching themes and patterns. From the quantitative data, averages and percentages were derived. Subsequently, the analysis of the qualitative and quantitative data was combined to redefine the strongest overarching themes and patterns. In addition, the qualitative data were used to provide an explanation for the outcomes of the quantitative analysis.

#### Health Care Providers and Professionals

After conducting the Web-based community study, in-depth interviews with HCPs were performed to understand their ideas and needs regarding an app, such as uGrow, and its insights. Independent HCPs were recruited for the study on the basis of their professional experience with parents of newborns and their interest in digital innovations and apps. The HCPs were compensated for the time they spent on study participation. Before the interview, a recruitment agency shared background information of the HCPs and sent all HCPs a high-level list of questions to prepare for the interview. The interviews were semistructured, including an intake and introduction, followed by a discussion about the meaning of a baby’s healthy development, the way in which HCPs support and advise parents in daily practice, and finally a discussion on the uGrow proposition, its features, and insights. The data gathered in the interviews were analyzed using a deductive, qualitative analysis method [27], where researchers analyzed the interview answers and defined overarching patterns.

**List of preliminary insights included in the in-home test.**

- Longest nap so far!
- This was the biggest bottle yet!
- You've tracked 3 hours of breastfeeding!
- You've tracked 10 hours of breastfeeding!
- First full night's sleep (eight hours consecutively)!
- You expressed a new record today!
- Wow, slept three nights in a row!

#### In-Home User Test

For the next study, a first version of the app, including a small set of preliminary insights (Textbox 1), was tested in an in-home user test. The aim of this test was to evaluate what parents think of the insights in the uGrow app and find out how insights might help parents in supporting their baby’s healthy development. The parents taking part in this study received compensation for the time they spent on study participation. The participants were asked to use the app at home for a period of 3 weeks. During the first 2 weeks, participants used the app in their own desired way (free usage). During the third and final week of the test, participants received a series of 5 tasks, for example, *In the next 24 hours, please track all of your baby’s feeds/naps*. After each of the 3 weeks, participants were asked to fill out a Web-based questionnaire containing both closed and open-ended questions.

The data collected in the in-home user test were analyzed using a deductive, qualitative analysis method [27], in combination with a quantitative analysis to derive averages and percentages.

### Development Phase

**Figure 1:**
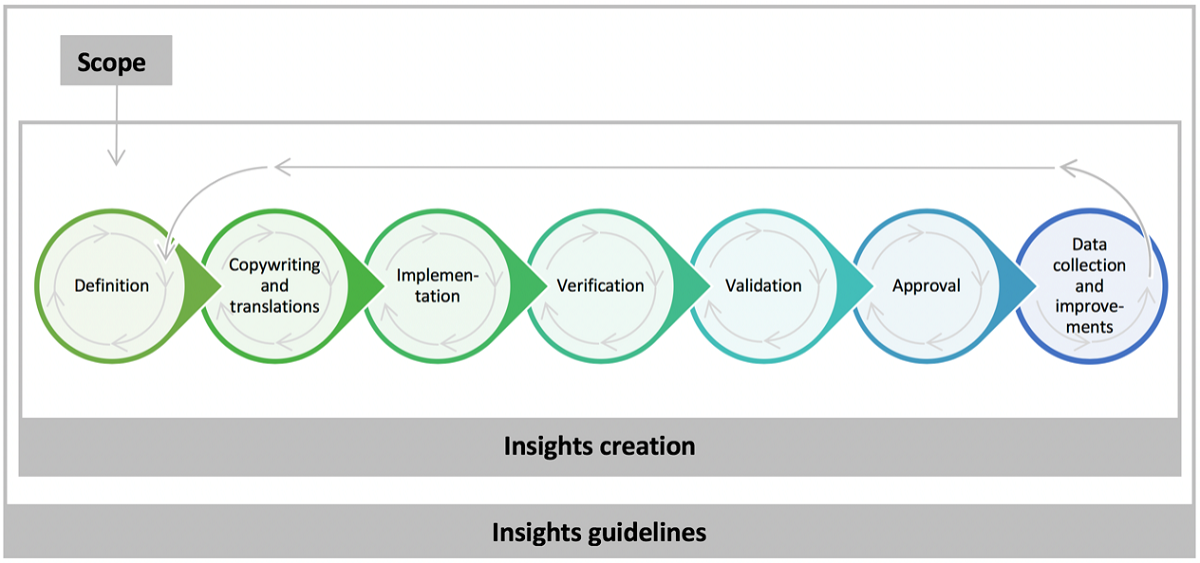
Insights development process.

The outcomes of the studies executed in the formative phase were used as input for the development of the insights. Before starting the actual development of the insights, we defined a process to ensure their controlled and systematic development. Through this process, we were able to guarantee the insights would be of high quality. Throughout the formative phase, we discovered that *quality* in the context of the insights is primarily rooted in 3 aspects. First, quality in terms of relevance, which addresses whether parents experience the topics addressed in the insights as being relevant. Second, quality in terms of appropriateness, which is about whether the insights fulfill a set of fundamental guidelines (eg, with respect to tone of voice, see section *Insights Guidelines*) to ensure success of the insights. Third, quality in terms of insights being evidence based, which means they are backed up by generally accepted guidelines or scientific evidence. To ensure this, we based insights on the general rule of thumb [8] that only information from scientific peer-reviewed literature, universities, volunteer organizations, and governmental organizations was used (eg, from United Nations Children’s Fund, the World Health Organization, La Leche League, AAP, and the National Health Service). Throughout each step in the insights creation process, we iteratively monitored whether the insight text and rules were still in line with scientific evidence or national/international guidelines. To address the 3 different aspects of quality, the development phase of the first batch of insights comprised the following 3 steps (Figure 1): (1) insights scoping, addressing the relevance of the insights by defining relevant topics and types of insights, (2) insights guidelines, addressing the appropriateness of insights through a set of fundamental and generic guidelines, and (3) insights creation, encompassing the criterion that insights are backed up with guidelines or scientific evidence and developed in a controlled manner. Each of these steps is elaborated below.

#### Insights: Scope

Scoping of the insights started with the selection of relevant topics. For the first version of uGrow, a limited number of topics were chosen for implementation. On the basis of the interviews with HCPs during the formative phase, feeding and sleep were concluded to be the most relevant topics for parents in the first months after the birth of their infant. Therefore, the first scope included the creation of insights on the topics of breastfeeding, bottle feeding, milk expression, and sleep. In addition, a small number of insights were added, which were related to other topics of the baby’s development: crying, weight, and temperature.

#### Insights: Guidelines

On the basis of the results from the formative phase, several aspects were identified, which may influence whether parents experience insights in a negative or positive manner. These results were used to define and develop insights guidelines that were followed during the insights creation process. They encompass a set of rules to ensure that the insights will be successful. An insight is considered successful when parents interpret the insight correctly (eg, parents understand the message in the insights as it was intended) and when parents think it is useful and reassuring for their personal situation with their baby. Some examples of the guidelines are not to judge parents and to respect the choices they make, provide multiple angles to a problem or issue, as every child is unique, and keep in mind that although insights are backed up by professional expertise and scientific research, they do not replace an HCP.

#### Insights: Creation

The topics selected in the insights scoping process were the starting point for the insights creation process. It comprised the steps defined below (though not always in the fixed order as described), and was adhered to always with the insights guidelines in mind (Figure 1).

##### Definition

First, literature searches were conducted into the topics defined within the insights scoping process. Subsequently, on the basis of the information found in the literature, ideation sessions were conducted for developing draft ideas for the insights. Each insight draft comprised a piece of text to be shown to the user, as well as a technical rule defining when the insight should be presented. In addition, for internal use and quality control, the (scientific) references for each insight were documented. In a second ideation session, the insights text and rule definitions (or specifications) were finalized by reviewing them against the insights guidelines. Then, all insights went through a preliminary check by a legal, regulatory, clinical, and marketing board to further ensure their quality. Once the insights passed this check, they were moved to the next step in the creation process: copywriting and translation.

##### Copywriting and Translation

This second step took place in tandem with implementation and verification. After defining them, the insights texts were rewritten by a professional copywriter and translated into the local languages of the markets in which the uGrow app would be available.

##### Implementation

The insights were implemented in the uGrow app by the development team.

##### Verification

In this step, the insights were verified to ensure they were implemented according to their specifications (eg, to check whether an insight text was shown correctly in the app).

##### Validation

A set of prototypical insights was validated against user experience to understand whether parents were satisfied with the defined insights. We validated whether parents experienced the insights as adding enjoyment, as being useful and reassuring, and as being motivational. Validation of the insights either took place after the definition phase, after copywriting and translation, or after the verification phase (see Figure 1 for these steps). When conducted after the definition phase or after copywriting and translation, validation comprised showing simple app prototypes (eg, simple screen versions) in a face-to-face user test and asking questions about the insights in these prototypes. When it took place after the verification phase, it comprised showing advanced, working app prototypes that included the insights, either in a face-to-face user test or in an in-home user test.

##### Approval

Insights were then formally documented, reviewed, and approved by the legal, regulatory, clinical, and marketing board before they could be released to the uGrow app in the field. For each insight, the documentation contained the final text of the insight, the technical rule specification, legal risk analysis, user risk analysis, validation results, and clinical substantiation.

##### Data Collection and Improvement of Insights

Once insights were live in the uGrow app in the field, they were monitored by collecting data mainly through but not limited to app analytics data and ratings and reviews. On the basis of the collected data, improvements for insights were defined and carried out.

### Summative Phase

In the summative phase, the first full set of insights was validated against parents’ experience through a postmarket survey in Germany. The uGrow app was launched in the Google Play Store and App Store in September 2016 in Germany and the United Kingdom. In this app release, 89 insights were included. After launch, a Web-based survey was conducted among German uGrow app users to validate parents’ experience with insights in general, with respect to enjoyment, reassurance, and usefulness, and whether insights increased parents’ motivation to use the app’s trackers for a longer period of time. In addition, a selected group of individual insights was validated to investigate whether parents experienced insights as adding enjoyment, as being useful and reassuring, and as being motivational. The German users were targeted during this test, as Germany is a primary market for Philips Avent. Through email, users were invited to fill out a survey about their experience with the uGrow app. The emails of the users were collected via the Philips Avent customer relationship management system (CRM). The data of the survey were then analyzed in a quantitative manner.

## Results

### Formative Phase

#### Web-Based Community

The Web-based community study took place in the United States and China, and it was executed in January 2015. The study was conducted in these countries because of the business interest of Philips Avent in these territories. Furthermore, previous internal Philips studies indicated American and Chinese parents being open to digital innovations for parenting.

##### Study Population

A total of 60 participants took part in the Web-based community, of which 30 American participants were living in the United States and 30 Chinese participants were living in China. A total of 7 American participants were in their third trimester of their pregnancy, and 23 participants were parents with a baby between 0 and 12 months old, of which 17 were first-time parents. A total of 9 Chinese participants were in their third trimester of pregnancy, and 21 participants were parents with a baby between 0 and 12 months old, of which 14 were first-time parents. All participants taking part in the study had shown an interest in the Philips Avent My Baby and Me app—the predecessor of the uGrow app. They were frequent app users, talkative, and had good writing skills.

##### Results

Participants from both countries highly valued the development of their child: they support their children in finding their own identity, in exploring their individuality, and in making their own choices. Overall, American parents more often emphasized their wish to provide love, safety, and trust as a safe basis for their child to start exploring life. Chinese parents often indicated they distance themselves from the authority-based parenting style of their own parents, and they emphasized independence in raising their child. In both countries, most parents took a broad and holistic perspective to the healthy development of their child. In addition to the physical health and growth of their child, parents also emphasized the importance of their child’s emotional and mental development. For example, on the one hand, parents thought about physical health and growth in terms of the child’s weight gain. On the other hand, parents thought about the child’s mood and whether they are achieving certain social-emotional milestones (eg, smiling, making eye contact). Tracking the physical aspects of the child’s development (sleep, feeding, and growth) was most important to parents in the first weeks or months after the baby’s birth. Parents used a wide range of tracking methods for this, such as taking paper-and-pencil notes, using excel files, and using forms provided by their HCP. Moreover, more and more often, parents used apps to track their child’s development. Parents discussed that their usage of existing apps around the topic of child development was mostly focused on tracking the psychical aspects and milestones (special moments) of their child’s development. Parents from both countries thought that the ability to track the general health, feeding sessions, and sleep/naps of the baby is an essential feature for a tracking app.

Parents in this study did not use apps to search for advice or information; they did not expect they could use an app for this. However, the Chinese parents in particular did use internet sources, such as blogs and Web-based communities. Parents perceived it as logical and relevant to have the app provide advice or information, that is, they thought it logical in the sense that an app in which they can track the baby’s development can also offer general advice and information about the baby’s development. In addition, they thought it relevant, as they could see how tracked data in the app can be used to provide them the right advice and information about their baby. Overall, in both countries, parents indicated they had a need for smart information on the basis of analysis of the data they tracked in an app, on the baby’s age and on the baby’s development phase. When Chinese parents were asked about their ideal app, they first mentioned their need for tracking their baby’s feeds, sleep, and growth, and then they mentioned their need for scientifically underpinned suggestions on the basis of data analytics. When American parents were asked the same question, they first mentioned the need for an easy-to-use solution, and then they mentioned the need for a solution that can track all important details of their baby’s development (feeding, sleep, and growth). In the third place, American parents indicated the need for intelligent feedback, advice, and predications on the basis of smart data analysis.

#### Health Care Providers and Professionals

Interviews with Dutch, German, and British HCPs were conducted in August 2015. The HCPs were recruited from these countries because of 2 reasons. The first was accessibility, as the Philips department conducting this research was located in the Netherlands. Dutch HCPs were easiest to recruit. Next to that, German and British HCPs were recruited because of a shifting interest of the Philips Avent business in the British and German markets. The interviews with Dutch HCPs were conducted in a face-to-face meeting, and the interviews with German and British HCPs were conducted via a Web-based video meeting.

##### Study Population

A total of 10 HCPs took part in the study, of which 7 were Dutch, 1 was German, and 2 were British HCPs. The HCPs had a variety of backgrounds (Table 1).

**Table 1:** Participants’ background and country.

Profession of health care professional	Country	Age	Sex
Pedagogue	Netherlands	48	Male
Pediatric nurse at the maternity ward in the hospital	Netherlands	56	Female
Pediatrician at the consultation office (*jeugdarts* in Dutch*)*	Netherlands	35	Male
Maternity caregiver at the consultation office	Netherlands	38	Female
Developmental psychologist	Netherlands	28	Female
Pediatrician at the consultation office (*jeugdarts* in Dutch*)*	Netherlands	35	Female
Pedagogue	Netherlands	42	Male
Pediatrician in the hospital	United Kingdom	46	Male
Pediatric nurse at the maternity ward and emergency unit in the hospital	United Kingdom	43	Male
Pediatrician in the hospital	Germany	42	Male

##### Results

First, overall, HCPs thought the uGrow app was relevant and useful to parents for keeping track of their baby’s development. They saw the uGrow app as a supportive tool for parents to understand, be reassured about, and support their baby’s development. They thought the app to be especially relevant for first-time parents of babies between 0 and 6 months old. In saying this, HCPs emphasized that the feeding and sleep sections in the uGrow app are most relevant for parents. HCPs explained that in the first months after their baby’s birth, parents will primarily ask questions about 2 topics: first and foremost, parents’ questions revolve around the baby’s feedings, and then the questions revolve around the baby’s sleeping behavior. Most HCPs saw some risks and limitations regarding the uGrow app, for example, some HCPs thought the uGrow app focused too much on only *measuring* the different aspects of the baby’s development and focused too little on the emotional and social aspects of the baby’s development. However, overall, HCPs were positive about recommending apps such as uGrow to parents, for whom they considered it appropriate and helpful. For example, a professional said the following:

[For] first time parents and parents unsure about the baby’s development,...I would recommend [it].

United Kingdom, Pediatrician

Most HCPs stated that they want to be sure of the quality of uGrow before recommending it:

If the app is scientifically underpinned, it can be interesting to recommend it to parents. When the app focuses on the fun aspect and makes e.g. predictions about the baby’s development, I would definitely not recommend this app.

Second, overall, HCPs supported the concept of insights for the uGrow app. HCPs considered specific types of insights as relevant, but they also considered some as irrelevant or risky. An insight such as “The average daily sleep time of baby is 14 hours 21 minutes” was considered relevant by most HCPs, and they did not see any risk or harm associated with insights of this type. HCPs had more mixed opinions about an insight such as “Congratulations, you have breastfed your baby for 10 hours already!” Some HCPs considered insights of this type relevant, as they are encouraging and rewarding:

This insight encourages and rewards mums who are breastfeeding their baby. I like this insight.

Other HCPs were not supportive of these type of insights:

This encourages mums to breastfeed their baby for more hours, that’s not something we want to encourage. It is better to say eg, 10 days of breastfeeding.

Some other HCPs viewed these type of insights as too game like, which, in their view, does not fit this type of app, and which might even make the app unprofessional. Most HCPs were supportive of insights, such as “Your baby has taken 500 ml today. HCPs recommend to feed a baby of Liam’s weight between 450 and 650 ml. So, Liam is eating well!” Some HCPs were critical toward the part in the insight that states “Liam is eating well,” as this might be a wrong conclusion, and they thought this conclusion should be drawn by the parent and not the app:

I think the app shouldn’t draw the conclusion “Liam is eating well,” I would leave this open for the parents to decide.

Other HCPs were more open to the app drawing a conclusion:

Drawing a conclusion can be good, to tell parents that everything is ok with the baby.

Some HCPs also considered it the responsibility of the app to inform parents when their baby is receiving too little or too much milk. In addition, some HCPs mentioned that they thought not all parents would be interested in professional guidelines, but they thought that parents might only be interested in whether their baby is happy with the feeding session. HCPs thought several elements are important for the insights in the uGrow app, for example, HCPs considered it important to clearly communicate to the parents what the target group is for the insights in the app—do the insights apply to preterm babies and sick babies? HCPs also considered it the app’s responsibility to provide potentially confronting information to parents, for example, when the baby’s development falls outside the norm. Some HCPs mentioned that it is difficult to predict how parents will interpret and experience an insight: “What if it isn’t going well with the parent or the baby?” Furthermore, they considered it important to thoroughly evaluate insights from multiple angles. HCPs considered it important that insights guide parents in interpreting the information provided and do not present a one-and-only truth. Another point mentioned by HCPs was about the importance of underpinning insights with scientific evidence and linking insights to articles with more detailed information. All HCPs indicated that insights should not replace an HCP, but insights should complement HCPs’ work. HCPs thought that in that way, insights can help educate parents and answer simple questions for which they would otherwise have contacted their HCP. HCPs also indicated that it is important to inform parents to contact an HCP when they are in any doubt about the information provided in the insights.

#### In-Home Use Test

The in-home use test was conducted in September 2015 in the Netherlands.

##### Study Population

In total, 49 Dutch parents were invited to participate in the study, of which 32 parents completed all phases of the in-home use test. Of these 32 parents, 78% (25) were mothers and 22% (7) were fathers. All parents had at least one child in the age range of 0 and 3 months. The parents were between 21 and 40 years old. Furthermore, all parents were already tracking 1 or more aspect(s) of their baby’s development at the start of the study.

##### Results

The majority of parents noticed they received insights (Textbox 1) that were based on data they had tracked in the uGrow app during the 3-week test period. The *longest nap so far* and *biggest bottle* insights were seen by two-third of the parents after 2 weeks of app usage. The other insights were seen by a smaller part of the parents. For example, a third of parents saw the *breastfeeding for 3 hours* insight after 2 weeks of usage, and a fifth of the parents saw the insight *full night’s sleep* after 2 weeks of usage. The majority of parents indicated that they experienced the amount and frequency of insights as good. Overall, most parents, 88% (28 out of 32), were satisfied with the insights. Parents gave 4 types of reasons for why they were satisfied. First, some parents indicated they experienced insights as personal:

The app understands me. It makes it very personal.

Second, parents indicated that they experienced insights as rewarding, supportive, and positive:

It feels as a kind of reward.

Lovely to read my child did something very well. I am not always aware of it in busy daily life.

Third, a part of the parents indicated experiencing the insights as motivating:

It makes you curious, what would the next insight notification be? Therefore, you keep on using the app.

Finally, some parents indicated that they were satisfied with the insights, as they helped them to become more aware of their baby’s development:

Some changes do not attract my attention because I’m always busy with my child. It can be difficult to see changes in feeding and sleep rhythm. That’s why I like the app pointing out small, but big changes.

Some parents, 9% (3 out of 32), were neither satisfied nor dissatisfied with the insights:

To me it doesn’t really add something, but I like reading them

The only reason I got the biggest bottle message was because I have difficulties entering the exact feeding amount in the tracker field

A small part of the parents, 3% (1 out of 32), was dissatisfied with the insights:

I received the notification “first time slept through the night,” this was the first time for the app, but since we have only started using this app after a couple of months, this happened much earlier already. The notification is not correct. Perhaps you can phrase it in a question: was this the first time sleeping through the night?

**Table 2:** Participants’ evaluation of individual insights (N=32).

Insight	(Very) positive^a,b^, n (%)	Neutral^a^, n (%)	Negative^a^, n (%)
Longest nap so far!	28 (88)	2 (6)	2 (6)
This was the biggest bottle yet!	23 (72)	7 (22)	2 (6)
You've tracked 3 hours of breastfeeding!	28 (88)	4 (12)	0 (0)
You've tracked 10 hours of breastfeeding!	26 (80)	6 (20)	0 (0)
First full night's sleep (8 hours consecutively)!	26 (80)	6 (20)	0 (0)
You expressed a new record today!	16 (50)	16 (50)	0 (0)
Wow, slept 3 nights in a row!	16 (50)	0 (0)	16 (50)

^a^Absolute number.

^b^Note that for the analyses we merged the categories *very positive* and *positive*.

A majority of parents, 70% (22 out of 32), indicated that they experienced insights as being motivational. A total of 26% (8 out of 32) of parents experienced the insights as neutral: neither motivating nor not motivating. A few parents, 3% (1 out of 32), experienced insights as not motivating. When evaluating the individual insights, at least half of the parents evaluated the individual insights as (very) positive (Table 2).

Furthermore, parents mentioned some novel ideas for the insights. For example, insights about milestones related to the baby’s age, insights about the time awake between naps, insights about the duration between bottles, insights about switching between breasts, and insights about how the baby is doing compared with the population average.

### Development Phase

In the first launch of the uGrow app, a total of 89 insights were implemented. They focused on babies in the age range of 0 to 3 months. They were primarily about feeding and sleep as parents, as mentioned before, generally have most questions about these topics in the first period after the baby’s birth. In addition, insights about crying, weight, and temperature were implemented in the uGrow app. The distribution of the number of insights over the different topics was as follows:

- 17 breastfeeding insights
- 20 bottle feeding insights
- 20 expressing insights
- 15 sleep insights
- 7 crying insights
- 2 weight insights
- 8 temperature insights

### Summative Phase

#### Study Population

After the first version of the uGrow app was launched, the Web-based postmarket survey was executed in Germany. Participants who created a uGrow app account and signed up for the Philips CRM program received an email invitation to complete the postmarket survey. Participants completed the survey on a voluntary basis. In total, a convenience sample of 54 German uGrow app users completed the full postmarket survey.

#### Results

Parents first answered a number of general questions about the insights: 85% (46 out of 54) of users reported to (fully) agree with the statement that insights add enjoyment to the usage of the uGrow app. A total of 11% (6 out of 54) of users reported to neither agree nor disagree with this statement, and 4% (2 out of 54) of users reported to disagree with this statement. A total of 94% (51 out of 54) of users reported to (fully) agree with the statement that insights are reassuring and useful. A total of 6% (3 out of 54) of users reported to neither agree nor disagree with this statement. A total of 77% (42 out of 54) of users reported to (fully) agree with the statement that insights motivate to continue tracking for a longer period of time. A total of 22% (12 out of 54) of users reported to neither agree nor disagree with this statement.

Furthermore, a subset of insights was evaluated individually. At least 78% (42 out of 54) of the parents liked all insights but one. In case parents did not answer they liked an insight, they were mostly neutral about it (neither liked nor disliked it), and only in a few cases did parent answer they disliked an insight. Overall, the percentage of parents that (very much) disliked an insight was between 0% and 3% (0 to 3 out of 54). Only in 1 case, 6% (3 out of 54) of parents (very much) disliked an individual insight. Table 3 shows the evaluation of the individual insights by parents.

**Table 3:** Participants’ evaluation of individual insights (N=54).

Insight	(Very) positive^a,b^, n (%)	% Neutral^a^, n (%)	% Negative^a^, n (%)
Your little one is almost 4 weeks old. It’s common for a growth spurt to happen around this time, so don't be surprised if you see a change in Sara’s feeding pattern.	51 (94)	3 (6)	0 (0)
Your baby’s temperature is on the high side for a newborn. Best to keep a close eye on his/her health, and consider checking in with your GP^c^.	49 (91)	3 (6)	2 (3)
Heads up: It’s actually completely normal for babies to wake up at night. This happens because their day and night rhythm is still developing. Over time, exposure to daylight and your own daily rhythm will help them to develop a sleep rhythm that’s close.	49 (91)	4 (7)	1 (2)
From your tracked feeds, we've noticed that your left breast is stealing the spotlight a little. Feeding evenly from both breasts can lead to a more stable milk supply, especially in the first few weeks.	46 (85)	8 (15)	0 (0)
Over the last week, you've tracked around 670 ml of bottle feeds per day. This is the recommended amount for babies between 4.5 kg and 5 kg.	43 (80)	10 (18)	1 (2)
Good news, your baby no longer has a fever. His/her temperature has gone down by 1.2 degrees since the last reading that you took today.	43 (80)	11 (20)	0 (0)
Heads up: Your baby may take longer than others to regain her birth weight because she was born on the larger side.	42 (78)	9 (16)	3 (6)
Around a month ago, your tracked bottle feeds averaged 490 ml per day. Now, you track on average 670 ml per day.	42 (78)	11 (20)	1 (2)
Did you know that three quarters of all mums express more milk from one breast? Based on your tracked sessions, you've expressed around 60 ml of milk from your left breast, and 40 ml from your right breast.	42 (78)	11 (20)	1 (2)
Nice work! You've recorded 3 hours of breastfeeding!	31 (57)	22 (41)	1 (2)

^a^Absolute number.

^b^Note that for analyses we merged the categories *very positive* and *positive*.

^c^GP: general practitioner.

## Discussion

### Principal Findings

The aim of this study was twofold. First, this study described the development process of high-quality insights for the uGrow app in the context of supporting parents with their baby’s healthy development. Second, this paper presented results of a user study about parents’ experiences with the insights and their opinions on whether these are supportive, insightful, and help reassure that their baby is developing in a healthy way.

#### Development of Insights

Before the actual development process of the insights, user studies with parents and HCPs took place to understand their needs within their contexts. Throughout the formative phase, we discovered that quality of the insights is primarily rooted in 3 aspects: relevance of the insights, appropriateness of the insights, and insights being evidence based. In this study, we described how these 3 aspects of quality can be embedded in a systematic and controlled development process, comprising scoping, guidelines, and a creation process. The insights scoping addressed whether parents experience topics covered in the insights as being relevant. The insights guidelines were about ensuring the appropriateness of the insights through a set of fundamental guidelines, which were kept in mind throughout the creation process. Subsequently, the insights creation process defined an iterative and step-wise approach to creating and implementing them. The insights creation process ensured that the texts and rules of the insights were evidence based. Throughout the insights creation process, from initial definition up to final approval, insights were iteratively reviewed and eventually approved of from multiple angles: user experience, clinical, regulatory, legal, and marketing.

#### Parents’ Experience

In the formative phase, a series of user studies were conducted. The results of the Web-based community showed that, first, when thinking about their ideal baby tracking app, American and Chinese parents have a need for smart advice and information on the basis of data analytics. Parents could see how data tracked in the app can be used to give them the right information about their baby. Subsequently, the results of the interviews with HCPs showed that they support the concept of insights; they can see insights as a supportive tool for parents for understanding and being reassured about their baby’s development. In the in-home user test, a preliminary set of insights were evaluated by Dutch parents. The results of this test showed that most parents, 88% (28 out of 32), were satisfied with the insights. Parents indicated 4 reasons for this: they experienced insights as personal, as rewarding, supportive and positive, as motivating, and as helpful in better understanding their baby’s development. After development and implementation of the first batch of insights, the postmarket survey was conducted as part of the summative phase. The results of this survey showed that German and British parents experienced the insights as adding enjoyment to the usage of the app (85%: 46 out of 54), as being useful and reassuring (94%: 51 out of 54), and as motivational (77%: 42 out of 54). When parents did not agree with a statement, they indicated they neither agreed nor disagreed with it. Overall, this shows that insights have the potential to support parents in their parenting role and positively impact app experience.

### Discussion of Findings

#### Quality and Quantity of Insights

As stated in the introduction, the quality of health information offered in apps is an issue, and this is increasingly brought forward by both medical professionals and users [28-32]. The development process for the insights we described in this paper presents a way in which the 3 essential aspects of quality—relevance, appropriateness, evidence-based—can be embedded in a systematic and controlled development process. The process presented here covers standard phases, such as definition, design, implementation, verification, validation, and maintenance, as well as phases described in known and industry-standard software development process models (eg, waterfall model, incremental model, V-model, and agile model). Furthermore, standard user-centered design principles have been applied while developing the insights. Both the parents’ and professionals’ perspectives were addressed by iteratively involving both user groups in the process. In this way, we could ensure quality of the content provided in the insights on all of the 3 quality aspects. With respect to quantity, the overload of health information on the Web has been identified as a serious problem [33]. Insights address this problem in 2 ways. First, the information in the insights is targeted, and it is thus more personal, as it is based on actual data of the baby that their parents have tracked in the app. Second, the information is more dosed, as insights only encompass a few lines of text and only link to an article with more in-depth information if necessary. Thus, in summary, insights in the uGrow app have been created in close collaboration with the target group, and they are relevant, appropriate and evidence-based, and tailored for the user. In this way, insights provide a solution to common issues with respect to quantity and quality found on the Web and in apps.

#### Follow-Up Questions

As summarized above, in our user studies, we found that parents think insights help them in better understanding their baby’s development. An interesting and relevant follow-up question is whether that understanding could contribute to parent-child bonding and attachment. From the literature, we know that (psycho-)education may improve bonding and attachment and reduce parental stress [34-36], and that that may improve parenting skills [37-39] and maternal sensitivity [38,40-42]. In addition, a positive association has been found between parental sensitivity and secure infant attachment [43-45], which in turn has been found to have positive physical, psychological, social, and cognitive long-term effects [46-49]. Taken together, one could argue that as insights contribute to parents’ improved understanding of their infant’s development, they may positively affect the development of bonding and attachment. However, as we did not collect information on these topics, follow-up research is required to investigate this hypothesis further. Another interesting question pertains to the personal nature of our insights. As it has become clear from the abovementioned, insights are intended to support parents in understanding their baby’s development and providing them with relevant information and education about that. To be able to do this, effective relay of the information provided in the insights is essential. Available literature shows that, in comparison with general communication, personalized communication stimulates active processing of information significantly more, and it thereby facilitates information adaptation [50-52]. From our postmarket survey, we learned that parents experience the insights as being personal. Given this, it can be argued that insights could present a more effective way than larger-scale communications to promote and support parents regarding the healthy development of their baby. Follow-up research by means of questionnaires and interviews could help answer the question whether information from the insights is indeed adapted more easily than that from mass communications.

#### Strengths and Limitations

This study knows a number of limitations. First, the findings from the validation studies described in this paper are based on Dutch and German parents, and they are thus not generalizable to other countries, especially not to nonwestern countries in, for example, Africa and Asia. Furthermore, the current insights have been based on globally accepted evidence, and they thus present a global solution. This means that the insights might not be tuned to local conceptions; therefore, insights might not be culturally sensitive. In addition, the sample size was relatively small, and the studies encompassed relatively short evaluation periods. A strong element of the findings in this paper is that they are based on multiple iterations and interactions with both parents and professionals. Furthermore, the insights are highly evidence-based and cover a wide variety of topics related to the infant’s development. In addition, as parents experienced insights as highly positive, the professional and parent perspective come together nicely in the insights.

### Conclusions

The uGrow app is one of the first apps that shows how insights based on tracked app data can be useful and reassuring for parents in supporting their baby’s healthy development and how they can add enjoyment to app usage. The insights development process that was followed shows how quality of the insights can be guaranteed in terms of insights being relevant, appropriate, and evidence-based, by following a systematic and controlled development process. Furthermore, the insights in uGrow provide an example of DDI by turning the tracked data of the baby into meaningful advice for parents, which may potentially support parent-infant bonding and attachment.

## Appendix

Multimedia Appendix 1 From left to right the uGrow tracker menu, the timeline including an insight, and a graph of tracked sleep data.

## Abbreviations

AAP: American Academy of Pediatrics
CRM: customer relationship management
DDI: data-driven innovation
HCP: health care professional
