# Supplementary figures and images for: Tracker-Based Personal Advice to Support the Baby’s Healthy Development in a Novel Parenting App: Data-Driven Innovation
Source: JMIR Mhealth Uhealth. 2019 Jul 24;7(7):e12666. doi: 10.2196/12666 (PMC6685129; doi:10.2196/12666)

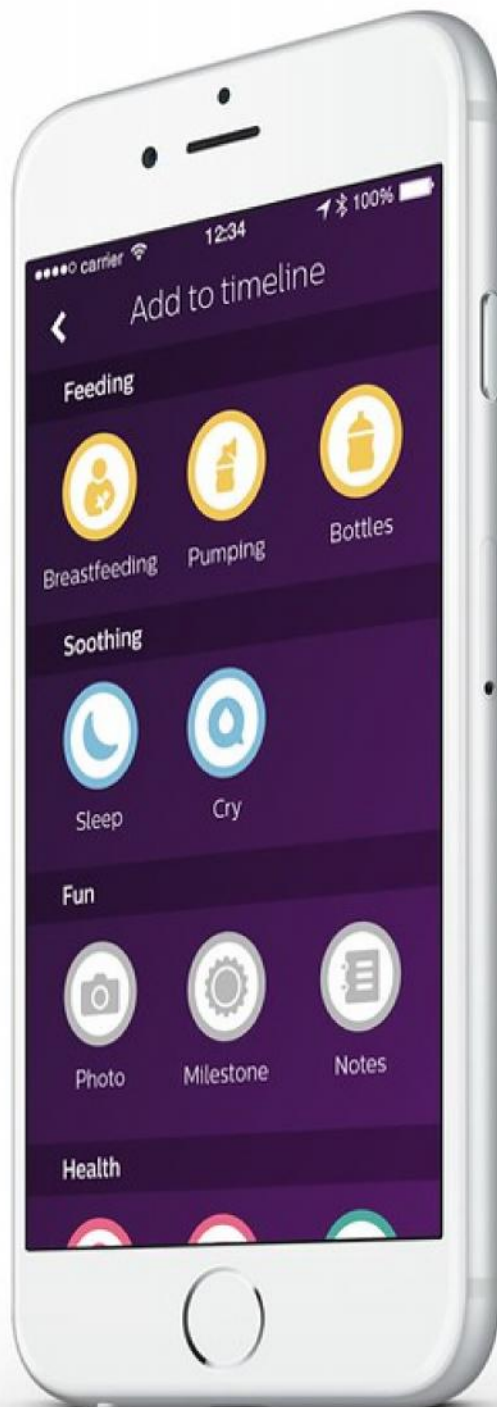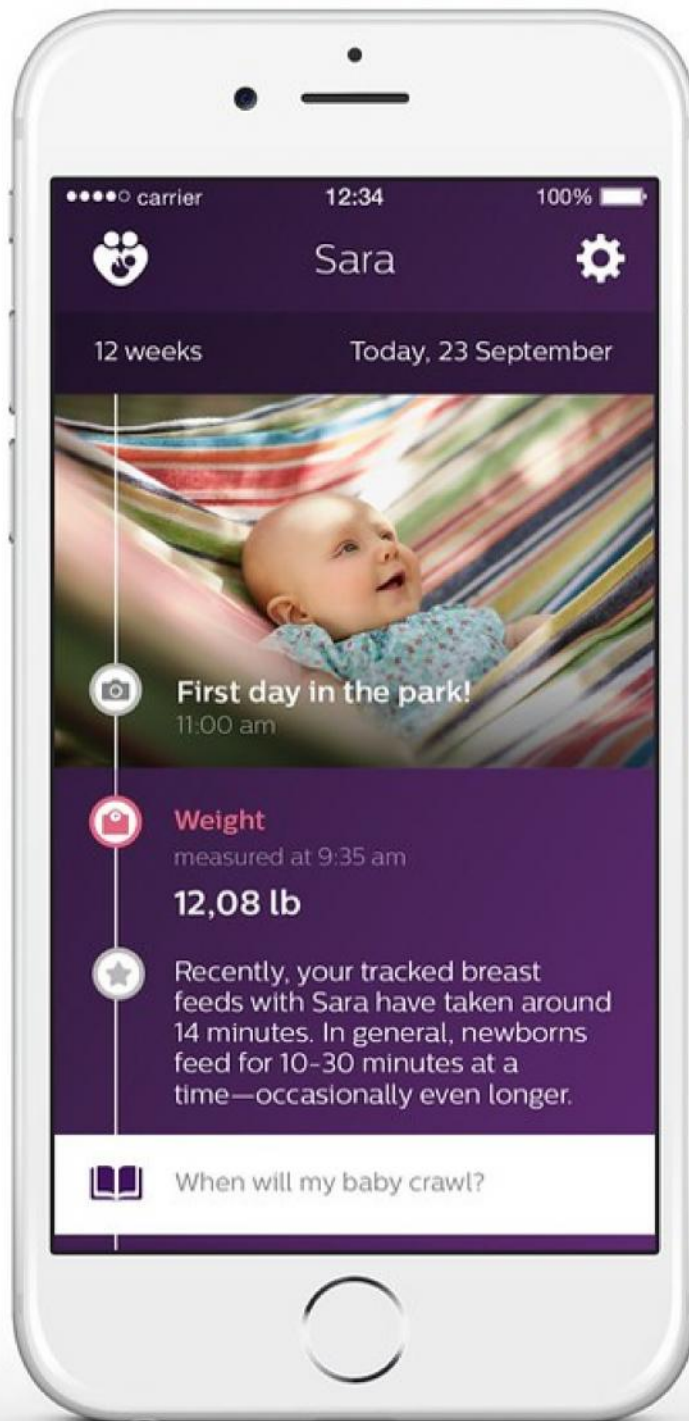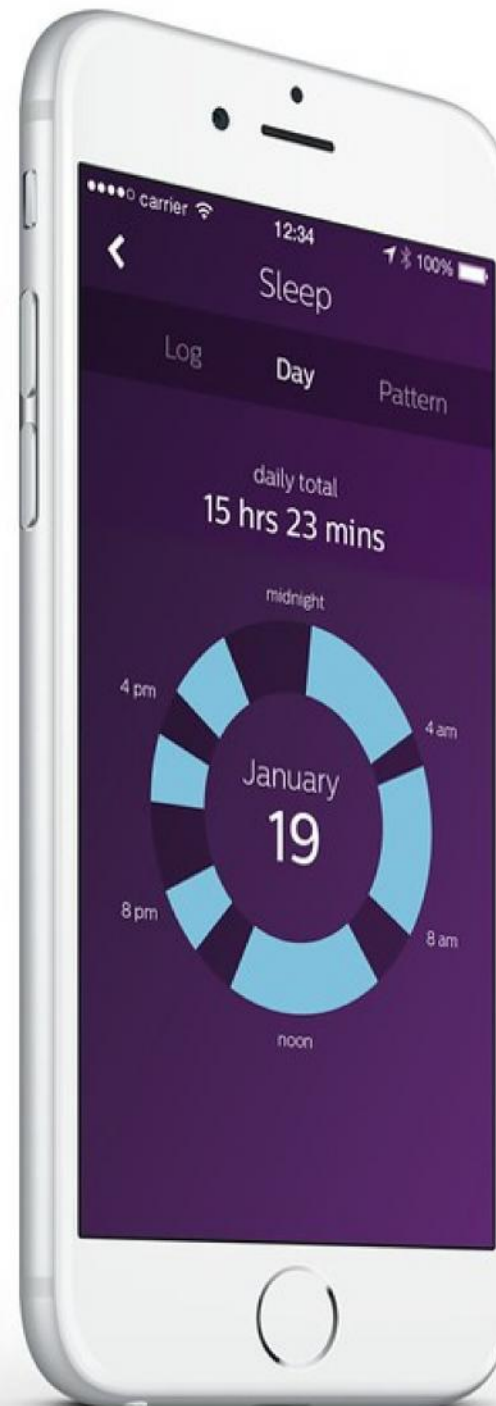

Supplement: Multimedia Appendix 1 [file mhealth_v7i7e12666_app1.pdf]
